# Implication of violence against women on not performing the cytopathologic test

**DOI:** 10.11606/S1518-8787.2018052000496

**Published:** 2018-11-05

**Authors:** Franciele Marabotti Costa Leite, Maria Helena Costa Amorim, Denise Petrucci Gigante

**Affiliations:** I Universidade Federal do Espírito Santo. Departamento de Enfermagem. Vitória, ES, Brasil; II Universidade Federal de Pelotas. Departamento de Nutrição. Programa de Pós-Graduação em Epidemiologia. Pelotas, RS, Brasil

**Keywords:** Battered Women, Violence Against Women, Domestic Violence, Intimate Partner Violence, Uterine Cervical Neoplasms, prevention & control, Papanicolaou Test, Health Care Quality, Access, and Evaluation, Mulheres Agredidas, Violência contra a Mulher, Violência por Parceiro Íntimo, Violência Doméstica, Neoplasias do Colo do Útero, prevenção & controle, Teste de Papanicolaou, Qualidade, Acesso e Avaliação da Assistência à Saúde

## Abstract

**OBJECTIVE:**

To analyze the association between intimate partner violence and not performing the cytopathologic test in the last three years.

**METHODS:**

It is a transversal study, performed in 26 health units in the city of Vitória, state Espírito Santo, from march to September 2014. The sample was constituted by 106 primary care female users, aging from 30 to 59 years-old. Data on cervical cancer screening were collected, besides the women’s sociodemographic, behavior, obstetric, and gynecological characteristics by an interview, and the World Health Organization recommended tool for identifying violence experiences was applied. The analysis was performed through the chi-square test for association, linear trend for ordinal variables, and the Poisson regression analysis with robust variance.

**RESULTS:**

Among the participating women, 14% (95%CI 12.0–17.2) had overdue Pap tests. Most women who did not perform the test had lower schooling levels, lower income, were smokers, in an unmarried union, having had their sexual debut before 15 years-old, three or more pregnancies, and two or more partners in the last 12 months. Women who suffered intimate partner sexual and physical violence were, respectively, 1.64 (95%CI -1.03–2.62) and 1.94 (95%CI 1.28–2.93) times more delayed in the Pap tests than non-victims.

**CONCLUSIONS:**

Violence is a significant exacerbating factor and affects women’s health negatively. Women who are physically or sexually victimized by their partners are more vulnerable to not performing Pap tests and, consequently, have fewer chances of early diagnosing cervical cancer.

## RESUMO

**OBJETIVO:**

Analisar a associação entre a violência por parceiro íntimo e a não realização do exame citopatológico nos últimos três anos.

**MÉTODOS:**

Estudo transversal, em 26 unidades de saúde do município de Vitória, ES, no período de março a setembro de 2014. A amostra foi constituída por 706 usuárias do serviço de atenção primária, com idade entre 30 e 59 anos. Foram coletados dados sobre o rastreamento do câncer de colo do útero, além da caracterização sociodemográfica, comportamental, obstétrica e ginecológica das mulheres por meio de entrevista e aplicado o instrumento recomendado pela Organização Mundial da Saúde para identificar a experiência de violência. A análise foi realizada por teste de associação do qui-quadrado, tendência linear para variáveis ordinais e análise de regressão de *Poisson* com variância robusta.

**RESULTADOS:**

Entre as participantes, 14% (IC95% 12,0–17,2) estavam com o exame de Papanicolaou em atraso. A maior prevalência de não realização do exame foi entre mulheres de menor escolaridade, em união consensual, menor renda, fumantes e com histórico de uso de drogas, coitarca antes dos 15 anos, três ou mais gestações e dois ou mais parceiros nos últimos 12 meses. Mulheres em situação de violência sexual e física cometida pelo parceiro íntimo apresentaram, respectivamente, 1,64 (IC95% 1,03–2,62) e 1,94 (IC95% 1,28–2,93) vezes mais prevalência de atraso no exame de Papanicolaou quando comparadas às não vítimas.

**CONCLUSÕES:**

A violência apresenta-se como um agravo importante e com impacto negativo na saúde da mulher. Mulheres vitimizadas, física ou sexualmente, por seus companheiros, estão mais vulneráveis a não realização do exame de Papanicolaou e, consequentemente, têm menos oportunidades de detecção precoce do câncer de colo do útero.

## INTRODUCTION

Cervical cancer is the fourth most common neoplasia among women in the world. About 528,000 new cases of it occurred in 2012, which were responsible for 266,000 female deaths, representing 7.5% of all female cancer deaths. Around nine in each 10 (87%) deaths due to this neoplasia happens in less developed regions. Mortality rates range from less than two per 100,000 in Western Asia, Western Europe and Australia, and to more than 20 per 100,000 in Melanesia (20.6) and Eastern Africa (27.6) ^1^ .

In Brazil, 16,340 new cases of cervical cancer were estimated for 2016–2017, a gross incidence of around 16 cases for each 100,000 women. It is the third most prevalent cancer, occupying the first position among women in the Northern region of the Country. It ranks in the second position in the Midwest and Northeast regions, while breast cancer is the most prevalent one, and the third and fourth positions for the Southeast and Southern regions, respectively ^2^ .

Since it is a public health issue, cervical cancer prevention strategies are essential. Vaccination against human papillomavirus (HPV), a risk factor for the disease development ^3^ , is a way of primary prevention, and the early diagnosis of injuries before they become invasive is highlighted as secondary prevention ^4^ .

The *Instituto Nacional de Câncer* (INCA – Brazilian National Cancer Institute) recommends, as a strategy for early detection, performing the cervical cytologic test, also known as Pap test, at every three years when there are two negative tests in an yearly gap. A sexually active woman should undergo the first test after 25 years-old and keep on performing it until she is 64. It may be interrupted when, after that age, the woman has at least two consecutive negative tests in the last five years ^5^ .

Not performing the Pap test is associated to the woman’s characteristics, such as: age, marital status, schooling levels, and income ^6^ ^,^ ^7^ . Cultural issues, such as: fearing pain, shame, not knowing the procedure, place where the test takes place, and being discouraged by the partner are also reasons for resisting ^8^ .

An epidemiologic case-control study showed that women with sexual violence history during childhood have less chances of performing the Pap test ^9^ . However, a population inquiry performed in the United States with women older than 18 points out that women who suffered intimate partner violence are more prone to cervical cancer screenings ^10^ .

The violence against women is an exacerbating factor in public health that affects a third of the world’s female population, and the intimate partner is the main perpetrator ^11^ . Researches show the impact of violence in the victims’ health, such as: cardiovascular and gastrointestinal disorders, and increase of pain and weariness complaints ^12^ ^,^ ^13^ . Besides, this phenomenon may be connected to not performing Pap tests ^9^ ^,^ ^10^ , one of the main strategies for cervical cancer early detection, which contributes to the reduction of incidence and mortality, as well as to physical, social, and psychological sequelae to the woman ^14^ .

This study had as it objective to analyze the association between intimate partner violence and not performing the cytopathologic test in the last three years.

## METHODS

This is a transversal study, performed in 26 Health Units (US) in Vitória, state Espírito Santo, which were covered by the *Estratégia de Saúde da Família* (ESF – Family Health Strategy) or by the *Programa de Agentes Comunitários* (PACS – Community Agents Program). The data collected from March to September 2014 referred to service users who had intimate partners during the 12 previous months to the interview. An intimate partner was defined as a partner or ex-partner, regardless of formal union, and current boyfriends, given that they were having sexual relations. For the outcome definition, we adopted the Brazilian Ministry of Health guidelines for performing the cervical cancer screening: two negative consecutive annual tests, with a repetition after three years ^5^ . The dependent variable “overdue cytopathologic test” was defined as not having performed the test in the last three years previously to the interview. Considering the recommended age for beginning the test of 25 years-old and the interval of three years after two negative results, 706 women aging 30 years-old or older, who could have the overdue Pap test, were elected for this study.

To identify the interest exposition, the experience of intimate partner violence (IPV) in the 12 months previously to the interview, the tool called “World Health Organization Violence Against Women” (WHO VAW Study) was used. This tool aims to discriminate the different forms of violence against women in their psychological, physical and sexual domains. It comprises 13 questions related to violence, capable of discriminating its different manners in diverse social contexts, being over-reaching and relatively short. It was validated for Portuguese and considered as having high internal consistency. Psychological, sexual and physical violence were considered present when the interviewed women answered yes to one of the tool’s items, and general violence was considered if she had experienced at least one of the three violence types ^15^ .

All the violence types were considered as exposition and its associations with the outcome. To estimate the sample size for a transversal study, we adopted a 95% trust level, with acceptable error of 5 percentage points, power of 80%, prevalence of exposed women (non-victims) who performed the Pap test of 0.75, and prevalence of non-exposed women (victims), who performed the Pap test, of 0.65, admitting a non-performance prevalence around 20% and a relative risk of approximately 1.2. The total population of 83,160 adult women who should use the municipality’s health units that had ESF or PACS was considered, with the population ranging from 858 to 12,189 according to the Health Unit. The sampling process was performed being proportional to the number of women registered in the Health Units, with a total necessary sample of 690 women.

Besides, to control the possible confounding variables, the sociodemographic characteristics were collected (age, race/color, schooling levels, marital status and family income), as well as behavior (alcoholic beverages ingestion, smoking, drug use history), and obstetric and gynecological ones (menarche age, current use of contraceptive methods, number of pregnancies, sexual debut age, and number of sex partners in the last 12 months).

The Pearson chi-square and linear trend tests were used in the bivariate analyses to investigate the possible association of confounding factors with the exposition and outcome, being the last one used only with ordinal variables. The robust Poisson regression was used in the analyses, registering the variables in the model if p < 0,20 for the bivariate analysis, by the regressive selection, and keeping them in the model if p < 0.05.

Two predictive models were used in the adjusted analyses: model 1 (adjustment for sociodemographic variables); and model 2 (adjustment for sociodemographic, behavior, obstetric and gynecologic variables). We used prevalence ratio and its respective trust intervals as an effect measure. The analyses were performed in State 13.0.

The study was approved by the Research Ethics Committee of the Universidade Federal do Espírito Santo (Statement 470.744). The research was explained to the eligible women and they signed the informed consent form. An individual interview was performed privately in the Health Unit by duly trained interviewers. At the end of each interview, the *Instituto Nacional de Câncer* (National Cancer Institute) folder, containing all cervical cancer prevention and screening promotion guidelines, was given to the interviewee, as well as a folder designed by the research authors containing the main care services for women victims of violence.

## RESULTS

The 706 women aging from 30 to 59 years-old who used the municipality’s Health Units that had ESF or PACS participated in the study. Most of them were 30 to 39 years-old (43.3%), declared themselves as brown (52.4%), and had studied for more than eight years (63.7%). Half of them were married and a third had a monthly family income above R$2,924,00. About 34% drank alcoholic drinks, 12% were smokers, and 9% had drug use history. Around 82% of them had their menarche until 14 years-old and 22% did not currently use contraceptive methods. Almost half interviewees had had three or more pregnancies. The first sexual relation of 54% of the women happened between the ages of 15 and 18 years-old, and more than 90% had at least one sexual partner in the last 12 months.

Among the interviewees, 14.5% (95%CI 12.0–17.2) did not undergo a Pap test in the last three years. The highest prevalence of not performing the cytopathologic test was seen on the women who had studied up to eight years, who declared being in a consensual union, belonged to the lower income group, had drug use history, and smoked. Higher prevalence of not performing the test was also observed in women who had three or more pregnancies, had their sexual debut before 15 years-old and had two or more partners in the 12 months prior to the interview (p < 0.05) ( Table 1 ).

**Table 1:** Sample characteristics and prevalence of overdue Pap test according to sociodemographic, behavior, obstetric, and gynecologic characteristics. Vitória, state Espírito Santo, March to September 2014. (n = 706)

Variable	n	%	In the last three years		

			%	95%CI	p
Sociodemographic					

Age (years)					0.456
30–39	306	43.3	16.3	12.6–20.9	
40–49	225	31.9	12.9	9.1–18.0	
50–59	175	24.8	13.1	8.9–19.0	
Race/Color^a^					0.662
White	156	22.9	14.1	9.4–20.5	
Brown	357	52.4	13.7	10.5–17.7	
Black	168	24.7	16.8	11.7–23.1	
Schooling level (complete years of study)					0.001
0–8	256	36.3	20.3	15.8–25.7	
More than 8	450	63.7	11.1	8.5–14.4	
Marital Status					0.011
Married	353	50.0	10.2	7.4–13.8	
Single/Dating	146	20.7	17.1	11.8–24.2	
Split/Divorced	20	2.8	15.0	4.8–38.4	
Consensual Union	187	26.5	20.3	15.1–26.7	
Family income thirds (Reais)					0.018^c^
Up to 1.500,00	227	32.1	17.6	13.2–23.2	
1.501,00 to 2.924,00	222	31.4	16.2	11.9–21.7	
Above 2.924,00	257	36.4	10.1	7.0–14.5	

Behavior					

Ingests alcoholic drinks (currently)					0.414
No	464	65.7	14.1	10.8–18.2	
Yes	242	34.3	16.5	12.3–21.8	
Smokes (currently)					0.027
No	507	71.8	12.4	9.8–15.6	
Yes	83	11.8	22.9	15.0–33.2	
Former smoker	116	16.4	17.2	11.4–25.3	
Drug use history					0.032
No	642	90.9	13.6	11.1–16.4	
Yes	64	9.1	23.4	14.6–35.4	

Obstetric and gynecologic					

Menarche age (years)					0.352
Until 14	577	81.7	13.9	11.3–16.9	
Older than 14	129	18.3	17.0	11.5–24.6	
Using contraceptive methods^b^					0.346
No	98	21.8	18.4	11.5–27.4	
Yeas	352	78.2	14.5	11.1–18.6	
Number of pregnancies					0.002^c^
None	54	7.7	7.4	2.8–18.3	
1–2	310	43.9	11.0	7.9–15.0	
3 or more	342	48.4	18.7	14.9–23.2	
Sexual debut age (years)					0.000^c^
Younger than 15	70	9.9	30.0	20.4–41.8	
15–18	381	54.0	14.4	11.2–18.3	
19 or older	255	36.1	10.2	7.0–14.6	
Number of sexual partners in the last year					0.001
1	639	90.5	13.0	10.6–15.8	
2 or more	67	9.5	28.4	18.8–40.3	

^a^ n = 681

^b^ n = 450

^c^ Tendency p value.

Higher predominance of overdue tests was observed among the women who suffered any type of IPV, when compared to the ones who did not report this experience ( Table 2 ).

**Table 2:** Prevalence of not performing the cytopathologic test in the last three years according to the type of violence suffered in the last 12 months. Vitória, state Espírito Santo, March to September 2014. (n = 706)

IPV in the last 12 months	n	%	95%CI	p
Psychological violence				
No	525	12.8	10.2–15.9	0.030
Yes	181	19.3	14.2–25.8	
Sexual violence				
No	661	13.5	11.1–16.3	0.004
Yes	45	28.9	17.5–43.8	
Physical violence				
No	643	12.9	10.5–15.7	0.000
Yes	63	30.2	20.1–42.6	
General violence				
No	508	12.2	9.6–15.4	0.007
Yes	198	20.2	15.2–26.4	

IPV: Intimate partner violence.

Women who were victims of IPV in the last 12 months, regardless the type of violence inflicted, had higher prevalence of not performing the Pap test in the last three years in relation to the ones who did not suffer aggressions in the gross analysis ( Table 3 ).

**Table 3:** Crude and adjusted analysis of the effects of intimate partner violence in the last 12 months, and not performing the cytopathologic test in the last three years. Vitória, state Espírito Santo, March to September 2014. (n = 706)

Violence in the last 12 months	n	Crude PR	95%CI	p	Adjusted PR 1	95%CI	p	Adjusted PR 2	95%CI	p
Psychological										
No	525	1.0		0.029	1.0		0.097	1.0		0.214
Yes	181	1.52	1.05–2.20		1.36^a^	0.95–1.97		1.25^b^	0.88–1.79	
Sexual										
No	661	1.0		0.003	1.0		0.015	1.0		0.046
Yes	45	2.15	1.30–3.53		1.82^a^	1.12–2.95		1.64^c^	1.03–2.62	
Physical										
No	643	1.0		0.000	1.0		0.001	1.0		0.002
Yes	63	2.34	1.53–3.58		2.10^a^	1.38–3.21		1.94^b^	1.28–2.93	
General										
No	508	1.0		0.006	1.0		0.034	1.0		0.078
Yes	198	1.66	1.15–2.38		1.48^a^	1.03–2.12		1.37^b^	0.97–1.94	

^a^ Schooling level, marital status, and income.

^b^ Schooling level, marital status, family income, smoking, drug use history, number of pregnancies, sexual debut, and number of partners.

^c^ Schooling level, marital status, number of pregnancies, sexual debut, and number of partners.

In the adjusted analysis for sociodemographic, schooling levels, marital status, and family income variables, being exposed to psychological violence did not remain associated to not performing the test (p = 0.097). This result was confirmed by the model 2, which included behavior, obstetric and gynecological variables as possible confounding factors for this association (p = 0.214) ( Table 3 ).

Regarding the implications of sexual and physical violence in cervical cancer screening, after the adjustment by model 1 (age, schooling levels, marital status, and income), a decrease in the gross average was observed, suggesting a positive confusion of these variables. In the final adjustment, a reduction in the effect measure was also observed, indicating a distortion of this association by the obstetric and gynecological variables. However, even after the control of all possible confounding factors, the association remained significant (p < 0.05), that is, women exposed to sexual and physical violence by their intimate partner had, respectively, 64% and 94% higher prevalence of not performing the Pap test in the last three years than the non-victim ones ( Table 3 ).

## DISCUSSION

14.5% users of health services in Vitória, state Espírito Santo, who were from 30 to 59 years old, have not undergone the cervical cancer preventive test in the last three years. This result resembles the finding of the *Pesquisa de Vigilância de Fatores de Risco e Proteção para Doenças Crônicas por Inquérito Telefônico* (VIGITEL – Vigilance Research of Risk Factors and Chronical Disease Protection via Telephone Inquiry). According to the study, in 2015, about 87% of women in Vitória performed the cytopathologic test in the last three years, that is, 13% did not undergo the test ^16^ . Such finding points out that the health services in the municipality have been reaching the goal stablished by the *Programa Nacional do Prevenção do Câncer de Colo do Útero* (National Program for Cervical Cancer Prevention), which proposes a cytopathologic test coverage of 80% to 85% of the female population, having a test for each three years ^5^ .

Despite the results suggesting that a significant percentage of women undergoes the Pap test according to the interval recommended by the Brazilian Ministry of Health, certain groups perform it less. This indicates the influence of some factors in the cervical cancer prevention actions.

The prevalence of not performing the Pap test in the last three years was associated to the woman’s conjugal situation. Single women presented higher prevalence of overdue tests in a research performed in Florianópolis, state Santa Catarina ^7^ . In our study, the higher percentage of not performing the test happened for women living with a partner, but unmarried, that is, who are in a consensual union. This result can be related to aspects as this women’s submission to their partners ^17^ , and also to the idea that a stable union stablishes an obstacle to the multiplicity of partners and, consequently, provides the woman with a certain degree of immunity to Sexually Transmitted Infections (STI), which is not true ^18^ .

Likewise this research, a study performed in Feira de Santana, state Bahia, has showed that women with lower schooling levels and more children have had higher prevalence of not performing the Pap test ^19^ . It was also observed a higher overdue test rates for women who has their first sexual relation before 15 years-old and more than two sexual partners in the year previous to the interview. This result is particularly concerning, given that the multiplicity of partners is connected to a higher risk for HPV infection, a necessary condition for developing cervical cancer ^20^ .

This is the first Brazilian epidemiologic study with users of the primary care that assesses the association between IPV and not performing the Pap test. The results are concerning and show that women who suffered IPV are more prone to having overdue Pap tests. Even after the control for possible confounding factors, it was verified, among sexual violence victims, a 64.0% prevalence of not undergoing the test in the last three years, while for the ones that suffered physical violence, the prevalence of overdue cytopathologic tests is almost twice as bigger (p < 0.05). Despite the non-association to psychological violence, 25% of victims of this kind of abuse have overdue cervical cancer prevention tests (p = 0.214).

It is important to interpret the presented estimates with caution, since this study’s data concern women who use primary care in a single Brazilian municipality, not representing the female population as a whole. Therefore, generalizing the results must be carried out cautiously. However, this must be considered, specially when analyzing the prevalence of outcomes and exposition, given that it is not necessary to perform a population-based study to investigate associations. Still, the possibility of a recall bias must be considered, since women tend to underestimate the time gone since they last performed the Pap test. However, self-reporting is widely used in national ^7^ ^,^ ^19^ and international ^10^ ^,^ ^21^ ^,^ ^24^ research, which were used for comparing and discussing the results in this study.

Studies on the association between violence against women and cervical neoplasia screening in Brazil were not found. Internationally, few are the published studies and those present methodological differences, which can justify the discrepancies between estimations and presented associations. A population-based American study with women aging from 18 to 45 years-old has shown that IPV was not related to performing the Pap test (p = 0.623) ^22^ . A research with 101 women diagnosed with breast, cervical, endometrial, or ovarian cancer has also showed no differences in cervical cancer screening for victimized and non-victimized women (p = 0.062) ^23^ . Likewise, a population-based study with Australian women did not find any significant association between violence against women and cervical cancer screening (OR = 1.18; 95%CI 0.99–1.40) ^24^ . The associations were close to the significance threshold in the last two studies.

In opposition, other studies have stablished a relation between being victimized and performing the test. Women who experienced physical or sexual violence, done by an intimate partner, had two times more chances of having performed the Pap test (OR = 2.05; 95%CI 1.26–3.31) ^10^ when compared to non-victims. Similarly, another research has showed that the chances of performing the Pap test were higher for women who suffered physical intimate partner violence (OR = 2.39; 95%CI 1.01–5.70) ^25^ .

Despite de methodology differences, specially regarding the measurement of violence, which is considered a complex event, the results in this study converge with the evidence reported by some authors ^9^ ^,^ ^21^ . Women users in the health service, aging from 40 to 74 years-old, who were victim of emotional abuse, when compared to the victims of physical or sexual abuse, had 87% less chances of having their Pap tests up-to-date (OR = 0.13; 95%CI 0.02–0.86) ^21^ . Women who were sexually abused before 18 years-old were also less prone to perform cervical cancer screening (35% *versus* 51%; n = 694; p = 0.009) ^9^ .

Although women exposed to violence access health services more frequently due to the physical and mental demands ^26^ , such access usually does not implies receiving the recommended preventive services ^22^ . Experiencing violence, specially sexual violence, may restrict the woman’s search for gynecologic appointments, due to shame of fear of retaliation by the partner, or even to the threat of unveiling the suffered violence. The gynecologic exam for victims, specially if performed coldly and carelessly, without explaining the procedure and its meaning, can become a moment of reliving the painful experience of the violence episode. Thus, instead of being a place of sheltering women, the gynecologic appointment can potentialize the feeling of fear and rejection towards the test ^4^ .

Besides the risk of physical injury and mental health damage ^12^ ^,^ ^13^ , the IPV, specially the sexual violence, makes women experience situations of frequently forced and unprotected sexual relations ^27^ . This makes the victim more prone to Sexually Transmitted Infections (STI) ^28^ , as the HPV, which is associated to a higher risk of cervical neoplasia ^29^ .

Still, the complex interaction among women under violence situation and the use of preventive services may depend on the type and severity of the experienced violence. All forms of violence have consequences that surpass the physical health sphere, also affecting the victims’ mental health. Such fact incapacitates selfcare and the care for others, possibly causing a diminished search for preventive health care ^4^ .

This study’s hypothesis was confirmed, since there was an association between not performing the cytopathologic test in the last three years and the experience of IPV. Therefore, given this context, and considering the impact of violence on the victimized woman’s health, health services have an important role on the promotion of preventive screening actions for cases of violence against women, as well as on offering preventive services. Professionals should gather efforts in order to reach these women and promote the opportunity for cervical cancer screening and for a comprehensive care, besides developing in these users the attitude of looking for health prevention and promotion services.
